# *HMGA1*-pseudogenes and cancer

**DOI:** 10.18632/oncotarget.7427

**Published:** 2016-02-16

**Authors:** Marco De Martino, Floriana Forzati, Claudio Arra, Alfredo Fusco, Francesco Esposito

**Affiliations:** ^1^ Istituto di Endocrinologia ed Oncologia Sperimentale del CNR c/o Dipartimento di Medicina Molecolare e Biotecnologie Mediche, Scuola di Medicina e Chirurgia di Napoli, Università degli Studi di Napoli “Federico II”, Naples, Italy; ^2^ Istituto Nazionale dei Tumori, Fondazione Pascale, Naples, Italy

**Keywords:** pseudogenes, HMGA, cancer, ceRNA

## Abstract

Pseudogenes are DNA sequences with high homology to the corresponding functional gene, but, because of the accumulation of various mutations, they have lost their initial functions to code for proteins. Consequently, pseudogenes have been considered until few years ago dysfunctional relatives of the corresponding ancestral genes, and then useless in the course of genome evolution. However, several studies have recently established that pseudogenes are owners of key biological functions. Indeed, some pseudogenes control the expression of functional genes by competitively binding to the miRNAs, some of them generate small interference RNAs to negatively modulate the expression of functional genes, and some of them even encode functional mutated proteins. Here, we concentrate our attention on the pseudogenes of the *HMGA1* gene, that codes for the HMGA1a and HMGA1b proteins having a critical role in development and cancer progression. In this review, we analyze the family of *HMGA1* pseudogenes through three aspects: classification, characterization, and their possible function and involvement in cancer.

## INTRODUCTION

The term “pseudogene” comes from the Greek word “pseudo” meaning false. Pseudogenes are also identified as “genomic fossils” [1]. They are outdated DNA sequences that lack protein coding ability because of the presence of frame shift mutations and early or delayed stop codons, even though they look like functional genes [2]. They are considered nonfunctional relatives of ancestral functional genes that might have lost their function during evolution [3]. Pseudogenes have been found in plants [4], bacteria [5], yeast [6], insects [7], nematodes [8] and mammals [9]. Based on their origins, pseudogenes have been classified into: (i) Processed pseudogenes - produced by mRNA retrotransposition [10]; (ii) Duplicated pseudogenes (called unprocessed pseudogenes) - originated from the duplication of functional genes that than become nonfunctional because of acquired mutations [10]; (iii) Unitary or Disabled pseudogenes - originated by mutations in the functional protein coding sequences [2].

Nowadays, pseudogene origin, evolution and function are only partially understood. The first paper about the biological role of a pseudogene was published about 16 years ago [11]. In fact, Korneev et al. reported that the neuronal nitric oxide synthase pseudogene worked as natural antisense in regulating neuronal nitric oxide synthase gene expression. However, recent studies have shown more functional roles for pseudogenes, associating them to long non-coding RNAs (lncRNAs) family [10, 12–14]. In fact, Poliseno et al. reported that *PTEN* pseudogene 1 (*PTENpg1*) is transcribed in human tissues and cancers and harbor microRNA (miRNA) response elements (MREs) for some of the same miRNAs that target its corresponding protein-coding gene, *PTEN* [12]. By sequestering miRNAs that would modulate *PTEN*, the corresponding pseudogene derepresses the protein-coding genes from miRNA regulation [12]. In this way transcripts could talk each other by competing for the same miRNAs, acting as competitive endogenous RNAs (ceRNAs) [15, 16]. In addition, Johnsson et al. characterized two *PTENpg1* antisense RNA isoforms, alpha and beta. The alpha isoform acts on the PTEN promoter inhibiting PTEN transcription by epigenetic mechanisms. On the contrary, the beta isoform directly interacts with *PTENpg1* RNA, which influences PTEN protein by changing *PTENpg1* stability and miRNA decoy activity [17]. Therefore, the overexpression of PTENpg1 sustains PTEN expression acquiring oncosuppressive functions [12].

Moreover, the human BRAF pseudogene (*BRAFP1*) has been recently found overexpressed in various tumor types, suggesting that it may contribute to cancer development. Karret et al. demonstrated the ceRNA role of both mouse *Braf-rs1* pseudogene (*Braf-rs1*)and its human ortholog, *BRAFP1*, eliciting the expression of BRAF and the activation of MAPK cascade both *in vitro* and *in vivo*. Indeed, miRNA bioinformatic analysis showed that murine *Braf-rs1* and *B-Raf* share 53 miRNAs, equally human *BRAFP1* and *BRAF* share 40 miRNAs. Thus, the *BRAF* pseudogene, *via* sequestration of common miRNAs, may work as a decoy for *BRAF* in mice and humans then upregulating *BRAF* and promoting MAPK signaling and tumorigenesis. Furthermore, mice overexpressing *Braf-rs1* develop an aggressive tumor similar to the human diffuse large B cell lymphoma. In addition, several transcriptional or genomic aberrations of *BRAFP1* were frequently found in multiple human cancers, including B cell lymphomas [18]. Taken together, pseudogenes are deeply involved in ceRNA hypothesis and give rise to large-scale controlling system across the transcriptome, critically increasing the functional data of human genome and acquiring main roles in physiological and pathological conditions [19].

Besides pseudogene-derived small RNAs have been demonstrated to have a role in chromatin repression [20]. Latest evidences show contribution of pseudogenes in regulating development and disease by encoding peptides or proteins [21–23]. Interestingly, Kandouz et al. detected the expression of *Cx43* pseudogene (*psiCx43*) in several cancer cell lines demonstrating its translationability in a protein of 43 kDa. Moreover, the psiCx43 protein overexpression was able to induce translational inhibition of Cx43 acting as a posttranscriptional regulator of Cx43, whose expression in cancer slows growth and renders the cells more sensitive to cytotoxic chemotherapeutics [23]. Finally, it has been reported that pseudogenes produce small interfering RNAs (siRNAs) in African Trypanosoma brucei and suppress several kinds of functional protein-coding genes through RNA interference pathway [24].

The family of High-Mobility Group A (HMGA) is composed of four proteins, HMGA1a, HMGA1b, HMGA1c, encoded by *HMGA1* gene at the end of alternative splicing, and HMGA2, encoded by the *HMGA2* gene [25]. *HMGA1* is located on chromosome 6p21 in humans and in the t-complex locus on mouse chromosome 17, whereas *HMGA2* is found on chromosome 12q13-15 in humans and at the *pigmy* locus on chromosome 10 in mice [26]. *HMGA1* and *HMGA2* genes are well conserved through the species, in fact, only few differences have been found between the human and the murine *HMGA* sequence [26]. They are non-histone chromosomal proteins, also identified as “architectural transcriptional factors” since they do not show a direct transcriptional activity, but modify the chromatin structure thanks to their DNA-binding domains, called “AT hooks”, by which they bind the DNA minor groove at AT-rich nucleotide sequences modulating the gene transcription [27–29]. HMGA proteins are expressed at low levels in adult tissues, but their expression is copious during embryogenesis [26], suggesting their important role in development. Indeed, the phenotypic study of *Hmga1* knock out mice showed that this protein has a critical role in different aspects of development [26, 30]. In particular, cardiac hypertrophy and type 2 diabetes were reported in *Hmga1*-null and heterozygous mice meaning that a correct quantity of HMGA1 protein is necessary for cardiomyocytic cell growth and regulation of the insulin pathway [26, 31–33]. In fact, the downregulation of HMGA1 protein leads to a reduced insulin receptor (INSR) expression in patients with insulin resistance and type 2 diabetes. The recovery of HMGA1 levels improved INSR gene transcription, restoring both expression of insulin receptor protein on cell surface and insulin-binding capacity [31].

It is worth noting that HMGA1 has been found abundantly expressed in all human neoplastic tissues analyzed, including, prostate [34–36], colon [37–39], breast [40–42], gastric [43–45], lung [46–48], testis [49–51], pancreas [52–54], ovary [55–57], thyroid carcinomas [58–60] and also in some forms of leukemia [61–63]. Importantly, HMGA1 expression level has been correlated with an advanced stage, occurrence of distant metastases and reduced survival in colorectal carcinomas [64–67]. To further sustain the HMGA1 function in cancer development, its expression levels have been associated with histologic grade of breast and ovarian carcinomas, where HMGA1 expression gradually enhances from no expression in normal breast tissue, to modest expression in hyperplastic lesions to overexpression in ductal carcinomas [67, 68], and augments from faintly expressed in ovarian carcinomas with low invasive potential to extremely expressed in invasive carcinomas [55, 69]. Importantly, HMGA overexpression plays a causal role in cell transformation. Indeed, their upregulation is able to transform rat fibroblasts [70] and human epithelial breast cells [71] and the block of HMGA1 protein expression prevented thyroid cell transformation induced by Kirsten Murine Sarcoma Virus, and it induced cell death into human thyroid anaplastic cell lines [72, 73]. Furthermore, the silencing of HMGA1 expression in colon cancer stem cells restores normal stem cell characteristics, reducing sphere-forming efficiency and recovering the asymmetric division pattern [39]. Finally, HMGA1 transgenic mice develop several benign or malignant neoplasias, such as GH/PRL-secreting pituitary adenomas, T-cell acute lymphoblastic leukemia and T/NK lymphomas [26, 37, 74].

The molecular processes involved in cell transformation induced by the *HMGA* genes are based on their capacity to positively or negatively control the expression of genes and miRNAs, small noncoding RNAs engaged in gene regulation [75, 76] and implicated in the regulation of cellular proliferation, invasion and apoptosis [77–79].

The upregulation of the *HMGA* genes in cancer may occur through oncofetal transcriptional mechanisms, which have not been elucidated yet. It is known that the high expression of HMGA1 in cancer cells needs a close cooperation between SP1 family elements and AP1 proteins, stimulated by the activation of Ras GTPase cascade [80]. Furthermore, recent studies have demonstrated the miRNAs HMGA proteins regulation by binding its 3′ untranslated region (UTR), provoking mRNA degradation or inhibition of its translation [81, 82]. In particular, several studies reported a strong HMGA regulation by miRNAs in pituitary adenomas (mir-15, mir-16, miR-34b, mir-214, miR-326, miR-432, miR-548c-3p, miR-570, miR-603 and mir-761) [83–85], in thyroid carcinomas (let-7) [86], in breast cancer (mir-26a, miR-33b) [87, 88]. Moreover, the loss of *HMGA2* 3′UTR, commonly found in benign tumors of mesenchymal origin, abolishes the inhibition of HMGA2 expression by several miRNAs [89, 90], leading to HMGA2 protein overexpression that accounts for neoplastic transformation.

## *HMGA1* PSEUDOGENES

The analysis of the human genome by bioinformatic database revealed the presence of eight processed *HMGA1* pseudogenes (*HMGA1Ps*): *HMGA1-p, HMGA1P1*, *HMGA1P2*, *HMGA1P3*, *HMGA1P4*, *HMGA1P5*, *HMGA1P6*, and *HMGA1P7* (Table 1).

**Table 1:** HMGA1 pseudogenes family

Gene	Location	Function	Main mutations	Reference
*HMGA1P1*	Xp21.3	Competitor protein for HMGA1 with different post-translational modifications.*	arg25, thr53, arg57, ser64	91-95,99
*HMGA1P2*	4q13.3	Competitor protein for HMGA1 with different post-translational modifications.*	arg57, arg59	94,99
*HMGA1P3*	12q24.11	Truncated form of HMGA1 with all molecular activities mentioned above.*	arg59, c-terminal tail deletion	94,96,97,99
*HMGA1P4*	9q34.11	-	-	-
*HMGA1P5*	10q22.2	HMGA1 non-homologous peptide*	-	-
*HMGA1P6*	13q12.12	Sustains the overexpression of several cancer-related genes by ceRNA mechanism	Stop codon	100-102
*HMGA1P7*	6q23.2	Sustains the overexpression of several cancer-related genes by ceRNA mechanism.	Start codon	100-102
*HMGA1-p*	2p13.2	Competes with *HMGA1* 3′ UTR for the binding to αCP1 RNA stability factor.	Few mutations	106

* The function of these pseudogenes still needs to be validated.

## *HMGA1P1* AND HMGA1P2

**Figure 1:**
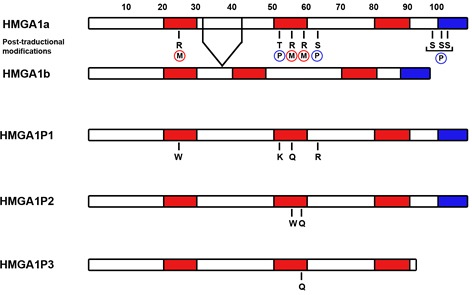
Structure of HMGA1Ps and their main mutations with respect to HMGA1 proteins Diagrams illustrating the domain structures of HMGA1a, HMGA1b, HMGA1P1, HMGA1P2 and HMGA1P3. Known post-translational modifications of human HMGA1a and HMGA1b proteins impaired in HMGA1Ps are highlighted (Phosphorylation in blue, methylation in red). The three AT-hooks are in red and the acidic tail in blue.

*HMGA1P1* and *HMGA1P2* pseudogenes, classified as processed pseudogenes, are located on Xp21.3 and 4q13.3 chromosome, respectively. They are not conserved during the evolution, but are only found in human genome. There are few mutations that distinguish *HMGA1P1* and *HMGA1P2* from *HMGA1a*. These changes in DNA cause few errors in protein sequence that importantly do not affect their translationability. Indeed, our preliminary data show that expressing vectors for *HMGA1P1* and *HMGA1P2* are able to code for proteins detectable by western blotting analysis. In fact, lysates from HMGA1-null cells transfected with the both vectors were positive to HMGA1 antibodies, which recognize the N-terminal aminoacids shared by HMGA1P1, HMGA1P2 and HMGA1. As shown in Figure 1, some HMGA1P1 and HMGA1P2 mutations hit aminoacidic residues that are frequently modified at post-translational level along the HMGA1 protein. Indeed, *HMGA1P1* is mutated at position 25 where a tryptophan residue substitutes an arginine, within the first AT-hook of HMGA1, which has been shown to be a major site of modification in tumor cells [91]. In fact, Sgarra et al. demonstrated that the arginine residue 25 is strictly related to the execution of programmed cell death in tumor cell lines [92]. *HMGA1P1* is also mutated at threonine residue 53 that is substituted with a lysine residue. Interestingly, threonine 53 was previously known as the main site of phosphorylation by cdc2 kinase during the cell cycle [93]. Compared with unphosphorylated protein, stoichiometric phosphorylation of recombinant human HMGA1 by cdc2 kinase strongly decreases the binding to DNA. Moreover, the HMGA1 protein arginine residue at position 57, along the second AT-hook, is replaced by a glutamine in HMGA1P1. It has been reported that PRMT6 methylates HMGA1 at the level of arginine 57, which is involved in the affinity for DNA binding and also in protein-protein interaction, thus implying an important role for arginine methylation in modulating HMGA functions [94]. Finally, HMGA1P1 brings a mutation at serine 64 where it shows an arginine residue. This is a Protein Kinase C (PKC) phosphorylation site [95]. Phosphorylation of HMGA1 by PKC resulted in a reduction of DNA-binding affinity as compared with that caused by the phosphorylation with cdc2 kinase, which phosphorylates threonine 53 [91, 95]. Therefore, HMGA1 could be additively phosphorylated by cdc2 kinase and PKC, and the resulting doubly phosphorylated protein exhibits a strong reduction in binding affinity [91, 95].

HMGA1P2 is mutated at arginine residues 57 and 59 where it shows a tryptophan and a glutamine residue, respectively (Figure 1). As well as arginine 57 methylation, it has been reported that HMGA1 arginine 59 is methylated by PRMT6 modulating its ability to bind to DNA and also the protein-protein affinity [94]. Therefore, if *HMGA1P1* and *HMGA1P2* pseudogenes coded for proteins, they could represent a sort of competitor proteins for HMGA1 wild-type with different post-translational modifications, altering HMGA1 properties in chromatin remodeling and protein-protein interactions.

## HMGA1P3

*HMGA1P3* pseudogene, only found in human genome, is classified as processed pseudogene and it is located on chromosome 12q24.11. Even though classified as non-coding RNA, it has only four aminoacidic mutations along the protein sequence compared to HMGA1 sequence, without affecting its translationability (Figure 1). Interestingly, arginine 59 is replaced with a glutamine residue as mentioned above for HMGA1P2 [95]. Moreover, *HMGA1P3* pseudogene lacks the C-terminal acidic tail that is a feature of HMGA proteins [26]. In fact, it has been revealed that HMGA1a and HMGA1b are phosphorylated by Casein kinase II (CK2) on three serines situated in the C-terminal tail (S98, S101 and S102) [96, 97]. Moreover, it is believed that the HMGA C-terminal tail may be important in modulating protein-protein interactions [97] and could be involved in enhancing transcription factor activity, but the role of these phosphorylations has been not completely uncovered yet [97]. Finally, expression of a truncated *Hmga1b* gene, without both the acidic tail and the 3′ UTR, significantly enhances growth rate and impairs adipocytic differentiation, also suggesting that the *Hmga1/T* mutant works in a contrasting manner [98]. Indeed, transgenic mice overexpressing the HMGA1 wild-type protein showed a reduction of the fat tissue in contrast with the obese phenotype of the *Hmga1/T,* mice even though there are no data that could explain why the wild-type and the truncated form of Hmga1 operate in opposite ways [98].

Given this scenario, if *HMGA1P3* pseudogene coded for protein, it could represent a truncated form of HMGA1 wild-type with all molecular activities mentioned above.

Interestingly, previous studies showed that *HMGA1P1, HMGA1P2* and *HMGA1P3* can be affected by chromosomal rearrangements in benign human tumors [99]. In particular, significantly higher frequency of chromosomal breaks within the chromosomal bands containing these pseudogenes were observed in uterine leiomyomas, lipomas, pleomorphic adenomas, and pulmonary chondroid hamartomas [99]. This study unveils the existence of an interesting pseudogene activation mechanism in tumor, since they could translocate, after chromosomal rearrangements, under a promoter region or within a functional gene, then coding for new fusion proteins.

However, no studies have evaluated *HMGA1P1, HMGA1P2* and *HMGA1P3* expression in human normal and malignant tissues where their possible deregulated expression might have consequences on the function of the wild type HMGA1 protein and then influence cancer progression.

## *HMGA1P4* AND *HMGA1P5*

The non-coding RNA *HMGA1P4* pseudogene is classified as processed pseudogene and is located on the human chromosome 9q34.11. Differently from the above mentioned pseudogenes, *HMGA1P4* genomic sequence shows low homology with *HMGA1*. Moreover, further bioinformatics analysis confirms its untranslationability. Therefore, it could not be classified either as ceRNA or as peptide related to HMGA1.

Another processed pseudogene related to *HMGA1* is *HMGA1P5*. It is present only in humans and located on the chromosome 10q22.2. As *HMGA1P4, HMGA1P5* has low homology along *HMGA1* sequence and it may code for a peptide not related to HMGA1 protein. At the moment there are no published studies about these pseudogenes.

## *HMGA1P6* AND *HMGA1P7*

**Figure 2:**
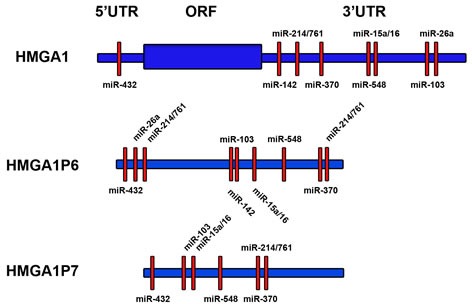
*HMGA1P6* and *HMGA1P7* mRNA sequence shares HMGA1-targeting miRNAs *HMGA1* (top), *HMGA1P6* (middle) and *HMGA1P7* (bottom) mRNA sequences are shown in blue. HMGA1-targeting miRNA seed matches (red boxes) within the high homology regions are shared among *HMGA1*, *HMGA1P6* and *HMGA1P7*.

The processed pseudogenes *HMGA1P6* and *HMGA1P7* are sited on 13q12.12 and 6q23.2 chromosome, respectively. They are not conserved through the evolution, but are present only in human genome [100–102]. These pseudogenes have high sequence homology with *HMGA1* both in the 5′ and 3′ UTRs and in the coding region (Figure 2). A missense mutation of the start methionine codon avoids *HMGA1P7* mRNA translation whereas *HMGA1P6* bears a mutation in the stop codon, which is postponed several aminoacidic residues downstream, producing a non-translatable mRNA [100–102]. In the homology sequences, among *HMGA1*, *HMGA1P6* and *HMGA1P7*, we retrieved conserved seed matches for miRNAs that have been predicted (miR-103, miR-142-3p, miR-370, and miR-432) or previously validated (miR-15 [83], miR-16 [83], miR-26a [103], miR-214 [104], miR-548c-3p [84] and miR-761 [104]) able to target the *HMGA1* gene (Figure 2).

**Figure 3:**
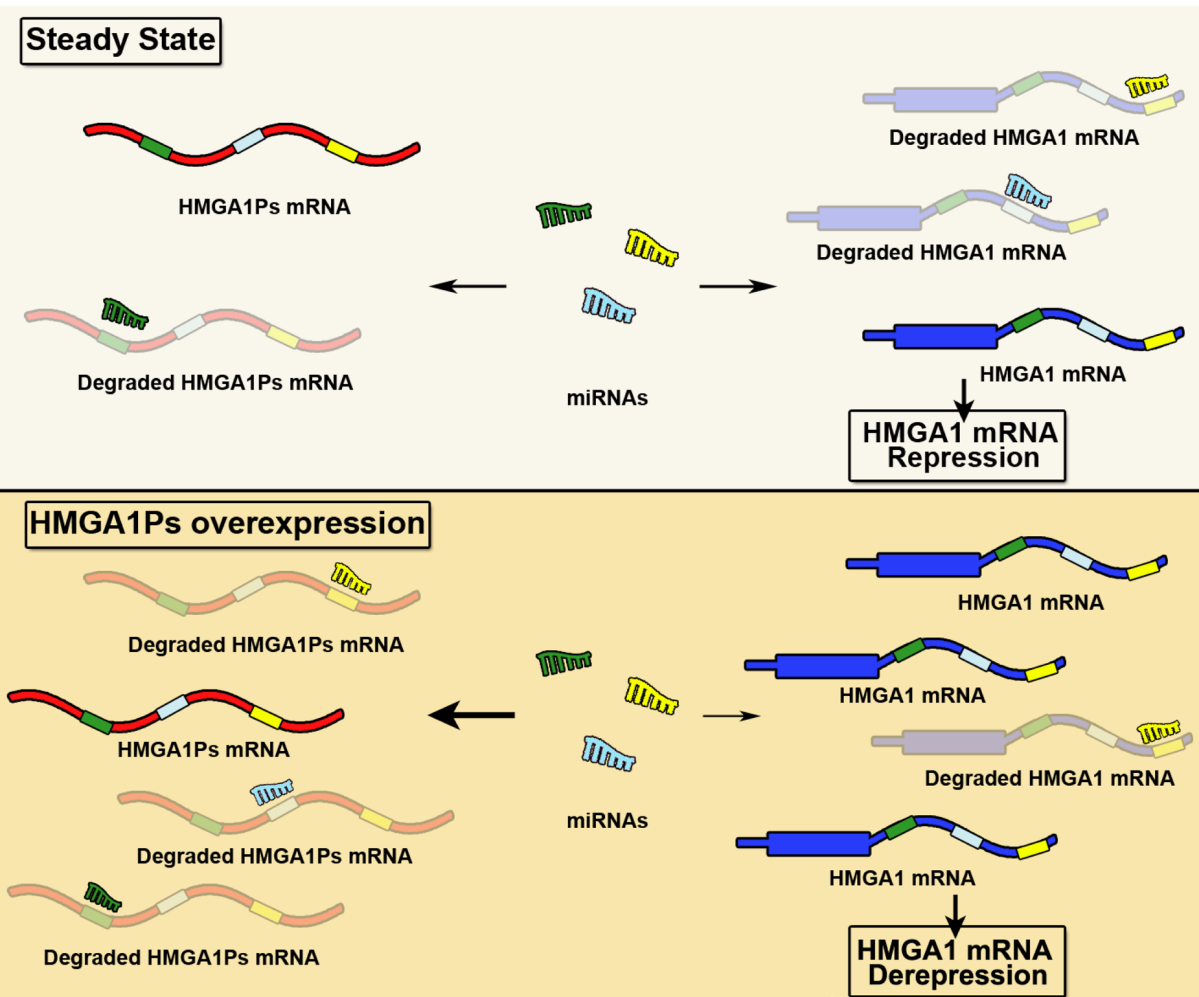
miRNA decoy function of *HMGA1Ps* In the steady state, equilibrium exists between the miRNAs and their targets *HMGA1* and *HMGA1Ps*. By contrast, the overexpression of *HMGA1P6* and *HMGA1P7* results in fewer miRNAs free to bind to *HMGA1*, and thus HMGA1 levels increase.

It has been reported that both *HMGA1P6* and *HMGA1P7* act as decoys for HMGA1-targeting miRNAs. In fact, their overexpression enhances HMGA1 protein levels whereas their knocking down results in the reduction of HMGA1 mRNA and protein amounts (Figure 3) [100–102].

Consistently, these *HMGA1Ps* have also oncogenic action by preventing apoptosis and enhancing cell proliferation and migration [100, 101]. Indeed, overexpression of *HMGA1P6* or *HMGA1P7* increases the growth rate and migration of different cell lines, contributing to tumor development [100–102]. Moreover, the generation of *HMGA1P6* or *HMGA1P7* transgenic mice confirms their oncogenic activity. In fact, mouse embryonic fibroblasts (MEFs) obtained from *HMGA1P6* or *HMGA1P7* [100] transgenic mice grow more rapidly and senesce later than their wild-type counterparts. Remarkably, in *HMGA1P6* and *HMGA1P7* overexpressing cells and MEFs we detected the upregulation of several cancer-related genes such as *High Mobility Group A2* (*HMGA2*), *Enhancer of Zeste Homolog 2* (*EZH2*), *Vascular Endothelial Growth Factor* (*VEGF*), and *Ephrin Type-A Receptor 3* (*Epha3*), with respect to the control cells [100]. This happens because of shared miRNAs targeting *HMGA1P6*, *HMGA1P7*, *HMGA1* and other cancer related genes. Therefore, high *HMGA1* gene or its pseudogene expression allows to increase other oncogene protein levels then contributing to cancer progression. Finally, a direct correlation among *HMGA1*, *HMGA1P6* and *HMGA1P7* expression in a group of human thyroid and ovary tumors has been shown [97–99]. Indeed, papillary thyroid carcinomas (PTC), which are fine differentiated and weakly aggressive, express low levels of *HMGA1P6, HMGA1P7* and *HMGA1*. On the contrary, anaplastic thyroid carcinomas (ATC), which are one of the most malignant human cancers, express very high *HMGA1Ps* levels that, moreover, correlated with HMGA1 protein levels [100]. Similar results were obtained in human ovarian carcinomas [100] and in endometrial carcinomas, where the *HMGA1P6* and *HMGA1P7* expression correlates with the malignancy rate. Interestingly, *HMGA1P6* and *HMGA1P7* were also overexpressed in human pituitary adenomas where the HMGA proteins play a critical role in their development [105]. In particular, *HMGA1P6* and *HMGA1P7* expression significantly correlates with *HMGA1* mRNA in somatotropic and nonfunctioning pituitary adenomas. Moreover, functional studies show that the enforced expression *HMGA1P6* and *HMGA1P7* enhances the proliferation of a pituitary adenoma cell line. Therefore, *HMGA1P6* and *HMGA1P7* overexpression contributes to keep high HMGA1 protein levels enhancing, then, its oncogenic ability.

## HMGA1-P

**Figure 4:**
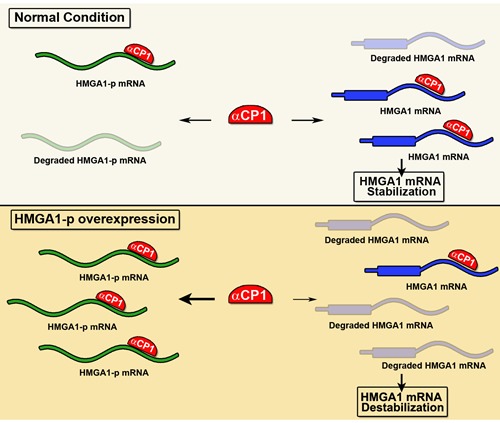
*HMGA1-p* function model In normal condition, the RNA-binding protein αCP1 stabilizes *HMGA1* mRNA by binding to its 3′ UTR. In diabetes, the *HMGA1-p* overexpressed transcript competes with *HMGA1* mRNA for the binding to αCP1, increasing the degradation of *HMGA1* mRNA.

*HMGA1-p* is located on chromosome 2p13.2. Its expression is able to induce destabilization of *HMGA1* mRNA [83]. Indeed, it has been demonstrated that the *HMGA1-p* RNA competes with *HMGA1* 3′ UTR for a critical RNA stability factor, the alpha C-binding protein (αCP1) [106]. The *HMGA1-p* was found overexpressed in diabetic patients then causing a significant destabilization of *HMGA1* mRNA with consequent loss of *INSR* expression, which is regulated by HMGA1, then generating the insulin resistance phenotype (Figure 4). Moreover, targeted knockdown of *HMGA1-p* mRNA results in an increase of *HMGA1* mRNA stability and expression levels, with a parallel correction in cell-surface INSR expression and insulin binding capacity [106]. Therefore, this study established a novel mechanistic linkage between *HMGA1-p* pseudogene expression and type 2 diabetes mellitus.

## CONCLUSIONS AND PERSPECTIVES

The mammalian genome contains an high number of pseudogenes (about 20,000 in humans) [107, 108] more than those present in other organisms. The biological meaning of pseudogenes was completely obscure until few years ago, whereas recent studies have shown their critical role in regulating gene transcription mainly functioning as decoy for miRNAs, and also evidenced a role of pseudogenes in carcinogenesis [109–116]. Interestingly, we have identified, by bioinformatic search, eight pseudogenes for the *HMGA1* gene whose expression is a feature of human malignancies with a key function in promoting cancer progression. From the analysis of the *HMGA1P* sequences it comes up that they could be able to regulate HMGA1 expression and function. Indeed, *HMGA1P6* and *HMGA1P7* act on the stability of *HMGA1* mRNA or by protecting them from miRNAs able to target this gene, whereas *HMGA1-p* competes with *HMGA1* 3′ UTR for a critical RNA stability factor, the αCP1. Conversely, HMGA1P1, HMGA1P2 and HMGA1P3 could represent a sort of competitor proteins for HMGA1 wild-type with different post-translational modifications, altering HMGA1 properties in chromatin remodeling and protein-protein interactions. So far, the role of *HMGA1-p* in type 2 diabetes and *HMGA1P6* and *HMGA1P7* in the progression of some human neoplasias appears well documented, but further analysis of their expression in embryonic and adult tissues, and in human carcinomas is required to be deeper investigated. Recently, it has been observed that transgenic mice overexpressing either *HMGA1P6* and *HMGA1P7*, develop lymphomas, infiltrating different organs likely working as ceRNAs for their oncogenic related genes.

Therefore, *HMGA1Ps* represent an epigenetic event, as well as miRNAs, able to regulate HMGA1 activity, and then play a critical role in all the processes such as cancer progression, development, metabolism and many other function in which HMGA1 is involved. The involvement of HMGA1 in all these important cellular processes likely accounts for the need of its fine regulation by using different molecular approaches. Interestingly, recent studies unveil a correlation between *HMGA1P6* and *HMGA1P7* and some clinico-pathological features, opening the perspective of using the evaluation of *HMGA1Ps* expression as diagnostic and prognostic marker, and maybe also in tumor classification. Therefore, the studies summarized here rehabilitate the *HMGA1Ps* from “junk” to a multifunctional pseudogene family that needs to be extensively studied.
